# Therapeutic efficacy of milbemycin oxime/praziquantel oral formulation (Milbemax®) against *Thelazia callipaeda* in naturally infested dogs and cats

**DOI:** 10.1186/1756-3305-5-85

**Published:** 2012-05-19

**Authors:** Bruna Motta, Manuela Schnyder, Fabrizio Solari Basano, Fabio Nägeli, Catherine Nägeli, Brigitte Schiessl, Egidio Mallia, Riccardo P Lia, Filipe Dantas-Torres, Domenico Otranto

**Affiliations:** 1 Veterinary Clinic, Via San Gottardo 128, CH-6828, Balerna, Switzerland; 2 Institute of Parasitology, Vetsuisse Faculty, University of Zurich, Winterthurerstr. 266a, CH-8057, Zurich, Switzerland; 3 Arcoblu s.r.l., Via Cardinale Mezzofanti 14, I-20133, Milan, Italy; 4 Novartis Animal Health Inc., Schwarzwaldallee 215, CH-4058, Basel, Switzerland; 5 Parco Regionale Gallipoli Cognato e Piccole Dolomiti Lucane, Basilicata, MT, Italy; 6 Department of Veterinary Public Health, Faculty of Veterinary Medicine, University of Bari, Str. Prov. Casamassima Km 3, I-70010, Valenzano, Bari, Italy; 7 Departamento de Imunologia, Centro de Pesquisas Aggeu Magalhães (Fiocruz- PE), Pernambuco, Recife, Brazil

**Keywords:** *Thelazia callipaeda*, Milbemycin oxime, Dogs, Cats, Treatment

## Abstract

**Background:**

Over the last few decades, canine and feline thelaziosis caused by *Thelazia callipaeda* eye worms has gained the attention of the veterinary community due to the spread of this ocular infestation in geographical areas previously regarded as non endemic. The therapeutic efficacy of milbemycin oxime/praziquantel tablets (Milbemax®) against *T. callipaeda* was tested in naturally infested dogs and cats.

**Methods:**

From January 2009 to July 2011 a placebo controlled and randomized field study was conducted in *T. callipaeda* endemic areas of Switzerland (CH) and Italy (ITA) involving client-owned animals. Dogs (n = 56) and cats (n = 31) were physically examined at enrolment Day 0 (D0) and twice afterwards (D7 and D14). Infested animals were orally treated with Milbemax® or with placebo tablets on D0 and, if an animal was found still infested with *T. callipaeda*, also on D7. On D14 nematodes were flushed from the conjunctiva, identified and counted.

**Results:**

Out of 56 dogs, 43 were included in the statistical analysis, whereas 13 were excluded because the products under investigation were not administered with food, as required by the label. On D7 and D14, 72.7% and 90.9% of treated dogs were eye worm free, whereas in the placebo group 95.2% and 76.2% still harbored nematodes, resulting in a mean percentage worm count reduction for the Milbemax® group of 86.1% and 96.8%, respectively. Both results were significantly higher (*p* = 0.0001) than the placebo group. Out of the 31 cats included in the study at D7 and D14, 53.3% and 73.3% treated with Milbemax® were free of *T. callipaeda*, while 81.3% and 73.3 in the placebo group were still harbouring eye worms, resulting in a mean percentage worm count reduction for the treated group of 62.2% and 80.0%, respectively. Both results were significantly higher (*p* = 0.0106 and *p* = 0.0043) than the placebo group.

**Conclusions:**

The commercial formulation of milbemycin oxime at the minimal dose of 0.5 mg/kg and 2 mg/k in dogs and cats, respectively, showed a high therapeutic efficacy in curing *T. callipaeda* infestations. The advantages of an oral application are additionally increased by the large spectrum of activity of praziquantel and milbemycin oxime against Cestodes and Nematodes infesting dogs and cats.

## Background

*Thelazia callipaeda* (Spirurida, Thelaziidae) is a nematode infesting the eyes of dogs, cats, rabbits, wild carnivores and humans [1]. This parasite has been commonly known as the “oriental eye worm” because of its occurrence, mostly in human beings, in far Eastern countries such as Thailand [2], China [3] and Japan [4]. Nowadays, it is evident that *T. callipaeda* is also endemic throughout Europe infesting domestic and wild carnivores in Italy [5,6] and Switzerland [7], and domestic animals in France [8,9], Germany [10,11], and Spain [12]. Importantly, human cases of thelaziosis in Europe have been recorded in Italy and France [13].

Since the incrimination of *Phortica variegata* (Diptera, Drosophilidae) as a vector of *T. callipaeda* in Europe under laboratory [14] and natural conditions [15], the knowledge on this nematode and its vector has been greatly enhanced. The adult whitish nematodes (about 0.5–2 cm) and first stage larvae (L1) localize under the third eyelid provoking lacrimation, conjunctivitis or even keratitis and corneal ulcer [16]. L1s are released by the adult worms into the conjunctival secretions of infested animals and they are ingested by *P. variegata* flies while feeding on animal eyes, developing into the infective third stage larvae (L3) within about 3 weeks [17].

The parasitic stages of *T. callipaeda* (i.e., adults and larvae) may be removed mechanically by rinsing the conjunctival sac with sterilized saline fluids or by collecting the adults with fine forceps or cotton swab; however, worm removal may be incomplete. Antiparasitic drugs, such as macrocyclic lactones (e.g., moxidectin) have been proven efficacious in treating thelaziosis by ocular instillation [18,19]. For compliance reasons it may be recommended to use systemic macrocyclic lactones licensed for dogs and cats, such as the spot-on formulation containing moxidectin (Advocate®, Bayer HealthCare AG) [20], or oral formulations containing milbemycin oxime (Interceptor®, Milbemax®, Program Plus®, Sentinel®, Novartis Animal Health) [21]. Interceptor® showed a good therapeutic and prophylactic efficacy in treating thelaziosis in naturally infested dogs [21].

Due to the increasing attention of pet owners and practitioners on canine and feline thelaziosis and to the spread of this ocular infestation in geographical areas previously regarded as non endemic, new therapeutic options are solicited. Thus, the aim of this work was to evaluate the therapeutic efficacy of a commercial oral formulation of milbemycin oxime/praziquantel (Milbemax® - Novartis Animal Health) in dogs and cats naturally infested with *T. callipaeda*.

## Methods

The efficacy of Milbemax® (Novartis Animal Health) was evaluated in a placebo controlled, multicentric, blinded and randomized field study conducted in Switzerland (CH) in the Mendrisiotto region (Southern Ticino, 101 km^2^, latitude 45°52 N and latitude 8°59 E, altitude ranging from 277–571 m above sea level), and in Italy (ITA) in the Basilicata region (Southern Italy, 9‘992 km^2^, latitude: 30° and 41°N; longitude 15° and 16° E, altitude ranging from 548–1367 m asl).

**Figure 1:**
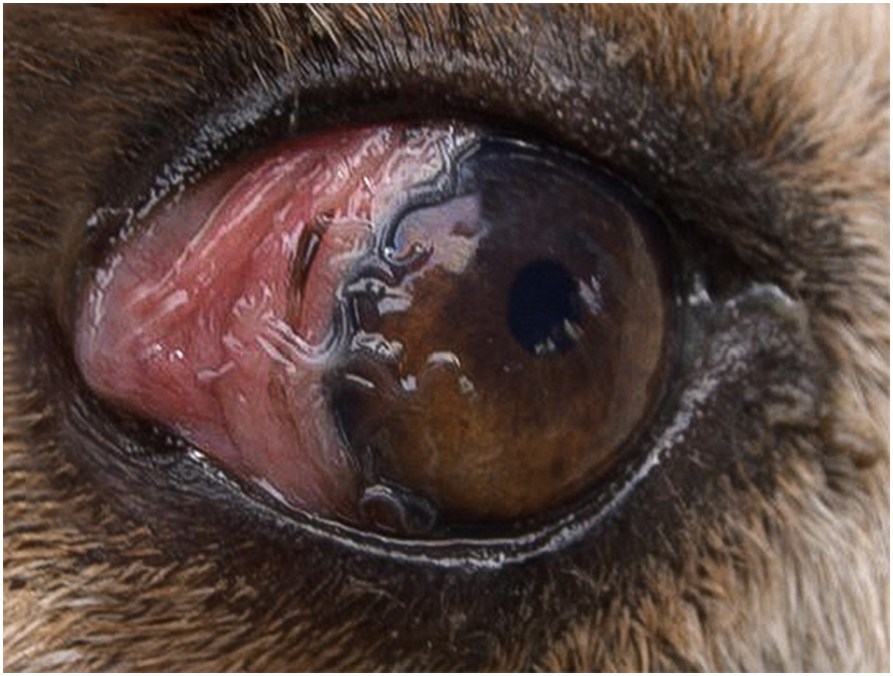
**Conjunctivits in a dog with*Thelazia callipaeda.*** Adult specimens of *Thelazia callipaeda* provoking conjunctivitis and mucopurulent discharge in the eye of a dog from Italy (Basilicata).

**Figure 2:**
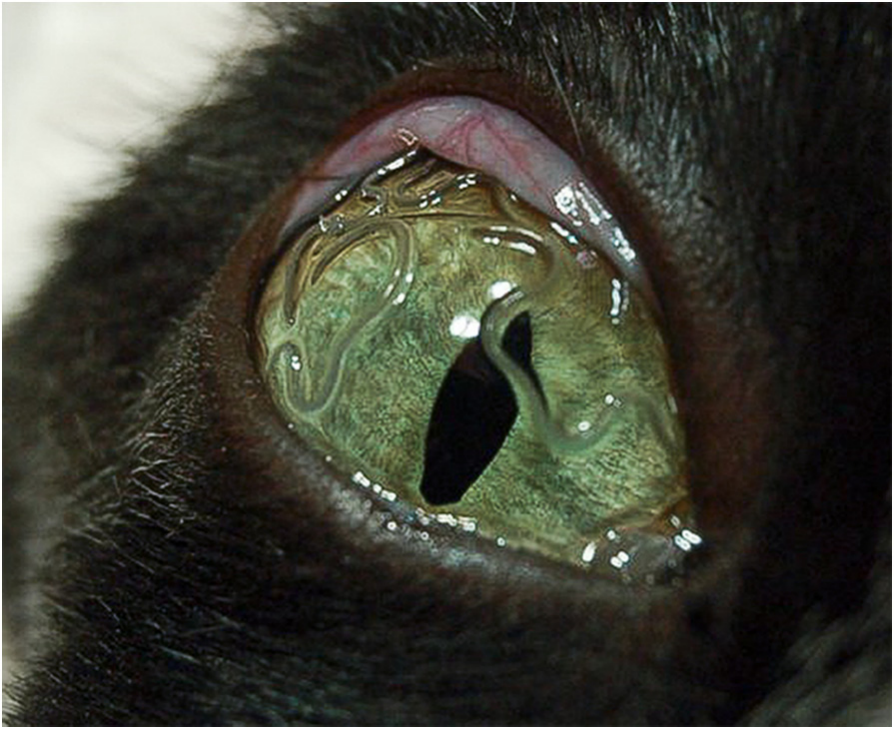
***Thelazia callipaeda*in the eye of a cat.** Eye of a cat from Basilicata infested with several adult specimens of *Thelazia callipaeda*.

Dogs (Figure 1) and cats (Figure 2) naturally infested with *T. callipaeda* were enrolled from January 2009 to June 2011 (CH) and from October 2010 to July 2011 (ITA). The study was conducted under Good Clinical Practice, according to EMEA VICH GL9, GL7 and GL19. The trial was performed after obtaining written animal owner consent, animal trial permissions of the Ticino cantonal (Switzerland) veterinary office (permission numbers 04/2009 and 05/2009) and of the Italian authorities (permission numbers MoH Italy n. DGSA 0018416-P-14/10/2010).

### Animals

All dogs and cats were client-owned, living in *T. callipaeda* endemic areas, of both sexes, various breeds, at least 6 weeks old and weighing 0.5 kg or more at time of inclusion. Animals were required to be infested with a minimum of one worm in one conjunctival pouch and living outdoors or regularly going outdoors. Testing for infestation of *Dirofilaria immitis* was done in Southern Ticino, being a heartworm endemic area, prior to inclusion in accordance with Milbemax® label content. Only *D. immitis* negative dogs were included in the study.

### Procedures

Dogs and cats were physically examined by the veterinarian at enrolment (D0) and then at two follow-up visits, (D7 and D14). A general physical examination was carried out at each visit to determine the health status of the animals. This examination also included body weight (b.w.) determination on D0 and D14. A blood sample was collected on day 0, before the treatment, for baseline haematology and clinical chemistry. In Southern Ticino the presence of *D. immitis* antigen (Dirocheck^(R)^, Synbiotics) and microfilariae (Knott’s test) was assessed in dogs. At each visit, both eyes were examined for the presence of eye worms by clinical inspection of the conjunctival pouch, including a thorough examination underneath the third eyelid using a cotton swab. If necessary, 2 drops per eye of a local anaesthetic (oxybuprocaine hydrochloride solution, Novesin® 0.4%, Omnivision) were applied into the conjunctival pouches. Clinical signs indicative of eye worm infestation (e.g., lacrimation, conjunctivitis, ocular discharge, keratitis, ulcers) were recorded and classified as absent mild, moderate or severe. A fluorescein test to diagnose ulcers was only performed if the animal was suspected to have ulcers. Worms were counted in each eye separately and the infestation intensity categorized into very mild (1 worm), mild (2–5 worms), moderate (6–10 worms) and severe (>10 worms).

On D0 dogs and cats were randomly allocated to treatment or control group by a random treatment allocation plan and orally treated according to body weight, following the label instructions of the commercial formulations of Milbemax® in both countries. In order to keep the blinding on the treatment details, even though both products (i.e., the milbemycinoxime/praziquantel and the placebo tablets) were comparable in appearance, a technician, different from the veterinarian that performed clinical evaluations, was responsible for the administration of the product and the storage of all test product related documentation.

On D7, if an animal was still found to be infested with *T. callipaeda*, a re-treatment with the same product at the same dose was administered. On D14, at the final visit, the conjunctival pouches were flushed with 5 ml of saline solution (0.9% NaCl) to collect larval stages of the parasite that were identified and counted, following centrifugation (5 min at 2000 g) and microscopic examination (40×).

During the study period animals were observed daily by their owners for health abnormalities and physically examined by the veterinarian in case of adverse events. If eye worms were still detected after D14, parasites were mechanically removed or animals were treated with an injectable solution of moxidectin 1 per cent (Cydectin®, Fort Dodge Animal Health) administered by ocular administration as previously described [18].

### Statistical analysis

Data were statistically examined using SAS® Version 9.2 (SAS Institute, Cary, NC, USA). Summary statistics including arithmetic and geometric mean, minimum, maximum and median were provided for all counts, percentages or continuous parameters of interest. Primary efficacy objective was to compare the Milbemax® group with the placebo group with respect to clinical cure (therapeutic efficacy), i.e., complete elimination of adult eye worms, seven and fourteen days after treatment. Secondary efficacy objective was to compare the treatment groups with respect to worm count reduction and reduction of severity and/or presence of clinical signs caused by eye worm infestation. Worm count reduction was calculated for each animal as follows: % reduction[t] = 100 × (WC[t_o_] – WC[t])/WC[t_o_], where WC[t_o_] = baseline worm count before treatment and WC[t] = worm count at time t after treatment. Fisher’s exact test was applied for the statistical comparison of clinical cure rates and infestation frequencies between treatment groups at different study days. Worm count reduction percentages were analyzed by analysis of variance methods if the assumption of normal distribution was satisfied on original scale or after transformation. Otherwise, Mann–Whitney *U* test was performed to compare the treatment groups. Adverse events observations were summarized and Fisher’s exact test was applied for the statistical comparison between groups. The level of significance was set at *p* = 0.05. Tests were performed two-sided.

## Results

### Dogs

Out of 56 dogs, which fulfilled the inclusion criteria, 43 (19 CH, 23 ITA) were included in the statistical analysis. Thirteen dogs were excluded because the investigational products (Milbemax® or placebo) were administered without any food, thus not as per the recommendations on the package leaflet. The included dogs were between 2 months and 13 years old, 26 were males (19 intact, 7 neutered) and 17 were females (12 intact, 5 spayed) and they were of various breeds (n = 24), including crossbreds (n = 19). Data on the worm count reductions are reported in Table 1. On D0, dogs treated with Milbemax® (n = 22) and dogs of the placebo group (n = 21) harbored a mean (arithmetic) of 6.14 and of 6.0 worms, respectively. The percentage of animals harbouring worms after treatment was significantly different (*p* = 0.0001) between the groups, in favor of the Milbemax® treated group. The mean percentage worm count reduction was significantly higher for the Milbemax® group than for the placebo group (*p* = 0.0001) on D7 and D14. The mean number of worms harbored by treated dogs was 1.36 and 0.14 on D7 and D14, respectively, while worm burdens of dogs of the placebo group were 5.71 and 5.38, respectively, with these differences being significant (*p* = 0.0001) at D7 and D14, but not at D0 (*p* = 0.4054). At D14 *T. callipaeda* larval stages were detected only in 3 dogs of the placebo group. Frequencies and percentages of clinical parameters indicative of eye worm infestation were not different between the groups.

**Table 1:** **Worm count reduction in dogs infested with*Thelazia callipaeda*after oral treatment with milbemycin oxime/praziquantel**

	Mean number of worms per dog (n, range)	Animals without worms (n, %)		Worm count reduction (%)	
	Study day 0	Study day 7	Study day 14	Study day 7	Study day 14
Milbemax® group (n = 22)	6.14 (1–22)	16 (72.7%)*	20 (90.9%)*	86.1%*	96.8%*
Placebo group (n = 21)	6.0 (1–22)	1 (4.8%)	5 (23.8%)	10.8%	27.5%

* significant difference between milbemycin oxime/praziquantel-group and placebo-group.

### Cats

A total of 31 cats (11 CH, 20 ITA) aging between 8 months and 14 years old, corresponded to the inclusion criteria and were included in the analysis. Of these, 19 were males (10 intact, 9 neutered) and 12 females (11 intact, one spayed). Data on the worm count reductions are reported in Table 2. At Day 0, treated (n = 15) and untreated (n = 16) cats harboured a mean (arithmetic) of 2.40 and of 2.38 worms, respectively. At D14, the number of animals free of *T. callipaeda* was higher in the treated group, with this difference being significant (*p* = 0.0268). The mean percentage worm count reduction for the Milbemax® group was significantly higher (*p* = 0.0106 and *p* = 0.0043) than the ones of the placebo group, on D7 and D14, respectively. The worm counts for the Milbemax® group were significantly lower than for the placebo group at D7 (0.93, *p* = 0.230) and 14 (0.80, *p* = 0.0129), while there was no significant difference on D0 (*p* = 0.4530). In 3 cats from the placebo group, larval stages could be found on D14. The presence of lacrimation on D0 was registered for 26.7% and 37.5% of the cats from the Milbemax® and the placebo group, respectively. This difference was not significant (*p* = 0.7043).

**Table 2:** **Worm count reduction in cats infested with*Thelazia callipaeda*after oral treatment with milbemycin oxime/praziquantel**

	Mean number of worms per cat (n, range)	Animals without worms (n, %)		Worm count reduction (%)	
	Study day 0	Study day 7	Study day 14	Study day 7	Study day 14
Milbemax® group (n = 15)	2.40 (1–11)	8 (53.3%)	11 (73.3%)*	62.2%*	80.0%*
Placebo group (n = 16)	2.38 (1–9)	3 (18.8%)	4/15 (26.7%)	20.0%	28.0 %

* significant difference between Milbemax®-group and placebo-group.

On D7 lacrimation was detected in 6.7% and 43.8% of the cats of the Milbemax® and of the placebo group, with this difference being significant (*p* = 0.0373), in contrast to data obtained on D0 (*p* = 0.7043).

## Discussion

The commercial formulation of milbemycin oxime (Milbemax®) at the minimal dose of 0.5 mg/kg and 2 mg/kg b.w. for dogs and cats, respectively, also containing praziquantel (5 mg/kg b.w.), showed a high therapeutic efficacy in curing *T. callipaeda* in naturally infested animals. In dogs the efficacy was 72.7% and 90.9% after a single or two treatments, at a weekly interval, both significantly differing from the placebo group. In cats, the therapeutic efficacy was 53.3% and 73.3% after a single or two treatments, at a weekly interval. It is known that praziquantel is not efficacious against nematodes. Since both actives of Milbemax®, milbemycin oxime and praziquantel, are well established on the market and known not to interfere with each other, it can be assumed that other formulations containing milbemycin oxime alone or in combination with other actives (e.g., Interceptor®, Sentinel®, Sentinel Spectrum® and Program Plus®; all Novartis Animal Health) may be efficacious against *T. callipaeda*. Furthermore, since *T. callipaeda* lives in the conjunctival pouches of the final host, an accurate dosing to ensure optimal blood concentrations of milbemycin oxime is needed to reach efficacious concentrations of the product in the conjunctives. This may explain the reasons for a higher efficacy of the product after a second treatment one week after the first, as also suggested by the results of a preliminary study on *T. callipaeda* naturally infested animals [21]. A lower efficacy observed on the 13 dogs (excluded from the statistical analysis) in which the treatment was administered without food, highlights the importance of a correct administration of the drug.

This study first evaluated the therapeutic efficacy of milbemycin oxime and praziquantel against *T. callipaeda* in cats. In addition, on the basis of a previous study in which the monthly administration of milbemyin oxime in dogs was highly effective (96.7%) for the prophylaxis of *T. callipaeda*[21], it may be argued that a similar prophylactic effect might occur in cats. This hypothesis deserves to be further tested under field conditions. Treatment of thelaziosis is an important issue in animals living in endemic areas, such as Basilicata in Italy (prevalence up to 60%; [6]), or Spain (prevalence of 39.9%; [12]) and Switzerland (prevalence up to 5%; [7]). An efficacious treatment against *T. callipaeda* is useful for pet owners considering the spread of the parasite in areas previously regarded as non endemic, such as France [9] and Spain [12].

The reasons for such an increase in cases of thelaziosis in dogs and cats throughout Europe are unknown, but it could be related to the spread of vector populations as well as to the occurrence of the infestation in wildlife species (e.g., foxes, wolves, beech martens and brown hares), which act as reservoirs for *T. callipaeda*[5]*.* Therefore, domestic animals which are traveling together with their owners from non-endemic to areas endemic for *T. callipaeda* should be treated since they are at risk of acquiring thelaziosis, as reported for some dogs in France or Germany [10,22]. Thus, monthly anthelmintic treatments, which are already recommended as a control strategy for dirofilarioses and other helminth infestations (e.g. see ESCCAP.org), should be considered for animals living in areas endemic for *T. callipaeda* in order to eliminate larval stages soon after their transmission from the drosophilid flies, thus interrupting the host-parasite transmission chain [17,23]. The high level of efficacy demonstrated in the current study suggests that further investigations should be carried out in order to test the effectiveness of the product when administered monthly during the risk season, in preventing *T. callipaeda* infestations in dogs and cats.

In addition, since Milbemax®-tablets for cats are flavor coated and chewable tablets are available for dogs, the oral formulations are very easy to apply, compared to the non-licensed local instillation of antiparasitic drugs [18,19] or to the mechanical removal of parasites from eyes. This especially applies when dealing with non-cooperative dogs and cats, where restraining them for manipulations around the eyes or even for spot-on applications is particularly difficult and bears the risk of trauma. Furthermore, wet coats or rainy days are reported to reduce the efficacy of topical administrations [20], a problem that is avoided by the drug administration *per os*.

## Conclusion

The commercial formulation of milbemycin oxime at the minimal dose of 0.5 mg/kg and 2 mg/kg milbemycin oxime for dogs and cats, respectively, showed a high therapeutic efficacy in curing *T. callipaeda* infestations. The advantages of an oral application of Milbemax® are additionally increased by the large spectrum of activity of praziquantel and milbemycin oxime against Cestodes and Nematodes infesting dogs and cats.

## Competing interests

The authors declare that they have no competing interests.

## Authors’ contributions

BM participated in the design of the study and in the field work, carried out the diagnostic assays and participated in the elaboration of the manuscript. MS and DO coordinated the study, participated in its design, in the field work and drafted the manuscript. FSB monitored the field studies, participated in the evaluation of the study results, helped to draft the manuscript and took the two pictures. FN, CN, RPL, FD-T and EM participated in the field studies sampling the animals. BS participated in the study design, coordinated the study participants and the statistical analysis and helped to draft the manuscript. All authors read and approved the final manuscript.

## References

[B1] Anderson RC Nematode parasites of vertebrates 2000 Their development and transmission. CABI publishing Guilford, UK 404 407

[B2] Bhaibulaya M Prasertsilpa S Vajrasthira S Thelazia callipaedaRailliet and Henry, 1910, in man and dog in Thailand AmJTrop Med Hyg 1970 19 476 479 10.4269/ajtmh.1970.19.476 5463089

[B3] Shi YE Han JJ Yang WY Wei DX Thelazia callipaeda(Nematoda: Spirurida): transmission by flies from dogs to children in Hubei, China Trans Royal Soc Trop Med Hyg 1988 82 627 10.1016/0035-9203(88)90535-4 3256118

[B4] Koyama Y Ohira A Kono T Yoneyama T Shiwaku K Five cases of thelaziasis British J Ophthalm 2000 84 441 10.1136/bjo.84.4.439c PMC1723424 10777285

[B5] Otranto D Dantas-Torres F Mallia E Digeronimo PM Brianti E Testini G Traversa D Lia RP Thelazia callipaeda(Spirurida, Thelaziidae) in wild animals: Report of new host species and ecological implications Vet Parasitol 2009 166 262 267 19782474 10.1016/j.vetpar.2009.08.027

[B6] Otranto D Ferroglio E Lia RP Traversa D Rossi L Current status and epidemiological observation ofThelazia callipaeda(Spirurida, Thelaziidae) in dogs, cats and foxes in Italy: a “coincidence” or a parasitic disease of the Old Continent? Vet Parasitol 2003 116 315 325 14580802 10.1016/j.vetpar.2003.07.022

[B7] Malacrida F Hegglin D Bacciarini L Otranto D Nageli F Nageli C Bernasconi C Scheu U Balli A Marenco M Emergence of canine ocular thelaziosis caused byThelazia callipaedain southern Switzerland Vet Parasitol 2008 157 321 327 18774229 10.1016/j.vetpar.2008.07.029

[B8] Dorchies P Chaudieu G Siméon LA Cazalot G Cantacessi C Otranto D First reports of autochthonous eyeworm infection byThelazia callipaeda(Spirurida, Thelaziidae) in dogs and cat from France Vet Parasitol 2007 149 294 297 17854998 10.1016/j.vetpar.2007.08.005

[B9] Ruytoor P Dean E Pennant O Dorchies P Chermette R Otranto D Guillot J Ocular thelaziosis in dogs, France Emerg Infect Dis 2010 16 1943 1945 21122226 10.3201/eid1612.100872 PMC3294570

[B10] Hermosilla C Herrmann B Bauer C First case ofThelazia callipaedainfection in a dog in Germany Vet Rec 2004 154 568 569 15144004 10.1136/vr.154.18.568

[B11] Magnis J Naucke TJ Mathis A Deplazes P Schnyder M Local transmission of the eye wormThelazia callipaedain southern Germany Parasitol Res 2010 106 715 717 19937259 10.1007/s00436-009-1678-4

[B12] Miro G Montoya A Hernandez L Dado D Vazquez MV Benito M Villagrasa M Brianti E Otranto D Thelazia callipaeda: infection in dogs: a new parasite for Spain Parasit Vectors 2011 4 148 21791108 10.1186/1756-3305-4-148 PMC3158752

[B13] Otranto D Dutto M Human Thelaziasis, Europe Emerg Infect Dis 2008 14 647 649 18394285 10.3201/eid1404.071205 PMC2570937

[B14] Otranto D Lia RP Cantacessi C Testini G Troccoli A Shen JL Wang ZX Nematode biology and larval development ofThelazia callipaeda(Spirurida, Thelaziidae) in the drosophilid intermediate host in Europe and China Parasitol 2005 131 847 855 10.1017/S0031182005008395 16336738

[B15] Otranto D Brianti E Cantacessi C Lia R Màca J The zoophilic fruitflyPhortica variegata: morphology, ecology and biological niche Med Vet Entomol 2006 20 358 364 17199746 10.1111/j.1365-2915.2006.00643.x

[B16] Otranto D Eberhard ML Zoonotic helminths affecting the human eye Parasit Vecors 2011 4 41 10.1186/1756-3305-4-41 PMC3071329 21429191

[B17] Otranto D Lia RP Buono V Traversa D Giangaspero A Biology of Thelazia callipaeda(Spirurida, Thelaziidae) eyeworms in naturally infected definitive hosts Parasitol 2004 129 627 633 10.1017/s0031182004006018 15552407

[B18] Lia RP Traversa D Agostini A Otranto D Field efficacy of moxidectin 1 per cent againstThelazia callipaedain naturally infected dogs Vet Rec 2004 154 143 145 14979443 10.1136/vr.154.5.143

[B19] Rossi L Rigano C Tomio E Frassetto D Ferroglio E Use of sustained-release moxidectin to prevent eyeworm (Thelazia callipaeda) infection in dogs Vet Rec 2007 161 820 821 18083983

[B20] Bianciardi P Otranto D Treatment of dog thelaziosis caused by Thelazia callipaeda (Spirurida, Thelaziidae) using a topical formulation of imidacloprid 10 % and moxidectin 2.5 % Vet Parasitol 2005 129 89 93 15817208 10.1016/j.vetpar.2004.12.020

[B21] Ferroglio E Rossi L Tomio E Schenker R Bianciardi P Therapeutic and prophylactic efficacy of milbemycin oxime (Interceptor) againstThelazia callipaedain naturally exposed dogs Vet Parasitol 2008 154 351 353 18456409 10.1016/j.vetpar.2008.03.011

[B22] Bussiéras J Chermette R Seillier A-M Quelques parasitoses canines exceptionelles en France. II - Un cas de conjonctivite parasitaire du chien, due àThelaziasp Prat Méd Chir Animal Compagn 1996 31 83 85

[B23] Kozlov DP The life cycle of nematodeThelazia callipaedaparasitic in the eye of the man and carnivores Doklady Akademy Nauk SSSR 1962 142 732 733

